# Injection of Matrix Metalloproteinase-9 Leads to Ventricular Remodeling

**DOI:** 10.1155/2022/1659771

**Published:** 2022-09-20

**Authors:** Enzheng Zhu, Congcong Yuan, Simiao Hu, Yiling Liao, Bowei Li, Yuliang Zhou, Wanxing Zhou

**Affiliations:** ^1^Department of Cardiology, The First Affiliated Hospital of Guangdong Pharmaceutical University, Guangdong Province, 510080, China; ^2^Metabolic Disease Research Center of Integrated Chinese and Western Medicine, Guangdong Province, 510080, China

## Abstract

**Objective:**

Previous studies have found that some ventricular remodeling is accompanied by increased matrix metalloproteinase-9 (MMP-9) in vivo, and MMP-9 inhibitors can reduce ventricular remodeling. However, there is still no direct evidence that MMP-9 causes ventricular remodeling. In this study, MMP-9 was injected into rats to observe whether MMP-9 caused ventricular remodeling, thereby providing direct evidence of MMP-9-induced ventricular remodeling.

**Methods:**

Forty-eight eight-week-old male Wistar rats were randomly divided, by weight, into control, low-, medium-, and high-dose MMP-9 groups and were administered normal saline or recombinant rat MMP-9 0.7, 1.4, or 2.1 ng/g, respectively, via intraperitoneal injection, twice per week. On the 28th day, six rats were randomly selected from each group (Stage I). The remaining rats continued receiving injections until the 56th day (Stage II). Echocardiography was performed to observe cardiac structure and function, and the left ventricular mass index (LVWI) was calculated. Myocardial pathological changes and the collagen volume fraction (CVF) were observed by HE and VG staining in myocardial tissue. MMP-9 levels in serum were tested using ELISA. Myocardial MMP-9 levels were measured using Western blots, and the myocardial expression levels of MMP-9 mRNA were assessed using RT-PCR.

**Results:**

During Stage I, serum MMP-9 and myocardial MMP-9 mRNA levels are increased; hypertrophic cardiomyocytes, disorderly arrangement of fibers, and endochylema dissolution are observed in the medium- and high-dose groups. The left ventricular weight index (LVWI) and myocardial MMP-9 increased, and the collagen volume fraction (CVF) reduced in the high-dose group. In Stage II, the left ventricular end-diastolic volume (LVEDV) and diameter (LVIDd) are higher, and CVF decreased in the medium- and high-dose groups. Myocardial pathological lesions intensified. Serum MMP-9 in the model groups and myocardial MMP-9 in the medium- and high-dose groups are increased.

**Conclusions:**

Injection of MMP-9 can lead to ventricular remodeling.

## 1. Introduction

Ventricular remodeling is a series of improper ventricle structural changes generated when the body deals with various cardiovascular damage types, including hypertension, cardiomyopathy, and myocardial infarction (MI). Ventricular remodeling includes myocardial parenchymal remodeling and myocardial interstitial remodeling [1]. Therefore, characterizing the mechanism of ventricular remodeling is a critical step toward overcoming cardiovascular diseases. The mechanism underlying ventricular remodeling involves the renin-angiotensin-aldosterone system (RAAS) and excessive sympathetic nerve activation. Outstanding clinical effects have been achieved with the targeted use of angiotensin-converting enzyme inhibitors (ACEI), angiotensin II receptor blockers (ARBs), and *β*-blockers. However, the inflexion point of ventricular remodeling control has not yet been identified, and the morbidity and mortality associated with cardiac failure continue to increase [2]. The global number of patients with cardiac failure increased to 23 million in 2020 [3], indicating a lack of understanding of the mechanisms underlying ventricular remodeling. Thus, further understanding of the mechanisms underlying ventricular remodeling is urgently required.

Matrix metalloproteinase-9 (MMP-9) is a member of the matrix metalloproteinase (MMP) family. In recent years, research evidence has indicated that MMP-9 is involved in the development of the fetal heart, angiogenesis, wound healing, and other physiological processes but is also associated with ventricular remodeling in various cardiovascular diseases [4]. For example, MMP-9 gene knockout or inhibition can significantly relieve ventricular remodeling in MI. MMP-9 inhibition can also mitigate the development of myocarditis and cardiomyopathy. Different ventricular remodeling processes in hypertension are accompanied by variations in myocardial MMP-9 levels [4, 5]. However, according to the “evidence chain” requirements for determining pathogenic factors, a specific factor is considered a pathogenic factor if (1) there is a significant increase in the incidence of the disease among individuals directly exposed to the pathogenic factor, and (2) inhibition of the factor can reduce or cure the disease. Therefore, this study injected MMP-9 into healthy rats to observe whether MMP-9 caused ventricular remodeling, aiming to provide direct evidence of MMP-9-induced ventricular remodeling. These findings provide a basis for further exploration of the mechanism underlying ventricular remodeling and the search for new drug therapeutic targets.

## 2. Materials and Methods

### 2.1. Experimental Animals and Grouping

In total, 48 Specific Pathogen-Free (SPF) level, healthy eight-week-old male Wistar rats (160-200 g) from the experimental animal centre of Southern Medical University were used in this study (certificate of quality number SCXX (Guangdong) 2011-0015). All animals were fed a conventional diet and housed in the SPF-level animal house of the First Affiliated Hospital of Guangdong Pharmaceutical University, with access to sufficient food and water. The rats were housed under a 12 h light/dark cycle, and the housing bedding was changed twice weekly. The experiment began after one week of adaptation. The rats were randomly divided into four groups according to body weight stratification: control, low dose, medium dose, and high dose (*n* = 6 in each group). The control group was given normal saline (2 ml), and the model groups were treated with 2 ml recombinant rat MMP-9 (rMMP-; purchased from the R&D systems Company, USA) at different doses through intraperitoneal injection twice per week: low-dose group 0.7 ng/body weight (g), medium-dose group 1.4 ng/body weight (g), and high-dose group 2.1 ng/body weight (g). By the end of the fourth week, six rats in each group were randomly selected into the Stage I group (*n* = 24), and modeling injections ceased for this group of rats. The remaining six rats in each group continued to receive the modeling injections until the end of the eighth week and were denoted the Stage II group (*n* = 24). The test parameters for each group were the same. This study was approved by the Animal Ethics Committee of the First Affiliated Hospital of Guangdong Pharmaceutical University (No. GYFY20161121010).

### 2.2. Blood Pressure Test

All rats underwent blood pressure measurements one day before the commencement of the experiments and at the completion (i.e., Stage I group on day 28 and Stage II group on day 56) under sober conditions. Blood pressure measurements were taken using an invasive caudal artery sphygmomanometer (ZH-HX-Z) (purchased from China Anhui Zhenghua Bio-instrument Company). Blood pressure measurements were performed uniformly after 10:00 am. During each measurement, the test table and sphygmomanometer were stored in the same static and windless environment, and the indoor temperature was maintained at 37°C. All rats were kept static for 5 min before blood pressure measurements.

### 2.3. Evaluation of Ventricular Remodeling

Rats were anaesthetized using 2% isoflurane at the end of either Stage I or II. The left ventricle structure and functions were examined using cardiac ultrasound (Philips HP5500), according to a previously described method [6, 7]. Cardiac ultrasound of all animals was performed by the same physician who was blind to the experimental grouping. Rats were then anaesthetized with sodium pentobarbital (50 mg/kg, intraperitoneally (i.p.)). The left ventricle was separated and weighed. Based on the weight, the LVWI was calculated according to the following formula: LVWI = left ventricle weight (LVW) (mg)/body weight (BW) (g). The left ventricular rat myocytes were cut into three parts (upper, middle, and lower) along the horizontal plane. The middle part was fixed in 4% paraformaldehyde and then underwent dehydration, the addition of transparency reagent, conventional paraffin embedding, and slicing (4 *μ*m). Tissue slices were submitted to HE staining. Myocardial cells and myocardial matrix fiber morphology and arrangement were examined under an HE staining microscope. Changes to myocardium collagen fibers were observed using VG staining (Baso Diagnosis Company, Zhuhai). Five visual fields without small coronary arteries were examined in each slice. The Image-Pro Plus color pathological graphic analysis system measured the collagen area. The collagen volume fraction (CVF) of the left ventricle was calculated as follows: CVF = collagen area of the left ventricle/measured area of view. Five CVFs were scored for each slice, and the mean was calculated.

## 3. Test of Serum MMP-9, Myocardial MMP-9, and Myocardial MMP-9 mRNA

### 3.1. ELISA

MMP-9 levels in serum were determined using ELISA. After the rats were sacrificed, the abdomen was cut, and the aorta abdominalis was separated. Blood was collected in a non-anticoagulant blood collection tube and kept static for 30 min. The blood samples were then centrifuged for 10 min at 4°C and 1500 rpm, and the serum was collected and placed immediately on ice. All samples were kept in a refrigerator at -80°C for later use, preventing repeated freezing and thawing. Serum MMP-9 was detected using a rat MMP-9ELISA kit (R&D Company, USA), according to the manufacturer's instructions.

### 3.2. MMP-9 Contents of Myocardium Detected Using Western Blot

Myocardial MMP-9 was determined using Western blots: 60 mg of myocardial tissue was collected and ground using liquid nitrogen to extract proteins for the bicinchoninic acid (BCA) protein assay kit (Beyotime Biotechnology, Haimen, China) quantification. Thirty micrograms of protein samples was electrophoresed and separated using 10% sodium dodecyl sulfate-polyacrylamide gel electrophoresis (SDS-PAGE). The proteins were later transferred to a polyvinylidene fluoride (PVDF) membrane. After soaking in milk for 45 min, the anti-rat MMP-9 antibody was added and diluted at a ratio of 1 : 1000 (Abcam Company, UK). The membrane was then incubated on a shaker at 4°C overnight. Following this, the membrane was cleaned with PBST for 30 min. The secondary antibody with horseradish peroxidase labelling was then added, diluted at a ratio of 1 : 5000 (Abcam Company, UK), and the membrane was incubated for one hour. The membrane was then cleaned again with PBST for 30 min. Finally, the reaction was strengthened with a chemiluminescence reagent. Samples were compressed and exposed using X-ray, and the images were analyzed.

### 3.3. Myocardial MMP-9 mRNA Detected by RT-PCR

Trizol was used to separate myocardial tissue and process the samples. Next, 1 *μ*l RNA was collected, and an ultraviolet spectrophotometer measured its optical density (OD) at 260 nm. The RNA concentration (*μ*g/ml) = OD260 × 40 × 100. The RT reaction of cDNA was completed with AMV reverse transcriptase. The RT-PCR system was run as follows: 2 *μ*l RNA, 4 *μ*l RNase free ddH_2_O, and 1 *μ*l Oligo dT, 72°C, 3 min. Later, the cDNA was cooled on ice, 5 *μ*l dNTP, 5 *μ*l RT buffer, and 1 *μ*l AMV reverse transcriptase were added and reacted at 42°C for one hour. The RT products were kept at -20°C for later use. The RT cDNA was used as the template, and primers were used for PCR amplification. The target gene primers were designed according to the literature and were synthesized by Sangon. The primers were as follows: for MMP-9 5′-CCTGGAGACCTGAGAACCAATC-3′, for MMP-9 Rev5′-CCACCCGAGTGTAACCATAGC-3′, for *β*-actin 5′-AAGGTCGCTCGGATGGTTAT-3′, and *β*-actin Rev5′-GGGAGTCCTCGTGGTAGTGATA-3′. The amplification program was 94°C 2 min; 94°C 30 s, 55°C 30 s, and 72°C 50 s for 30 cycles, followed by 72°C 3 min. The PCR products were tested using 2% agarose gel electrophoresis (AGE).

### 3.4. Statistical Analysis

SPSS 21.0 data analysis software (IBM, Armonk, NY, USA) was used to analyze the data statistically. Normality and homogeneity of variance tests were first carried out. Each group's normally distributed measurement data were represented by the mean ± standard deviation ($\bar{x}\pm \text{SD}$); nonnormally distributed data were represented by the median. Intergroup comparisons were performed using a one-way analysis of variance (ANOVA) under the assumption of homogeneity of variance. The LSD test was used for pairwise comparisons. Nonparametric tests were used for data that violated the assumption of homogeneity of variance. The Pearson test was used for correlation analysis. Values of *p* < 0.05 were taken to indicate a statistically significant difference.

## 4. Results

### 4.1. No Difference in Baseline Values; Experimental Survival Rate 100%

The survival rate of the rats in the model and control groups was 100%. There were no significant differences among the groups in terms of mean body weight and baseline blood pressure level (*p* > 0.05) (Table 1).

### 4.2. Injection of MMP-9 Can Cause Changes to Ventricular Remodeling

#### 4.2.1. Injection of MMP-9 Can Increase LVWI as a Function of Time and Dosage

**Figure 1:**
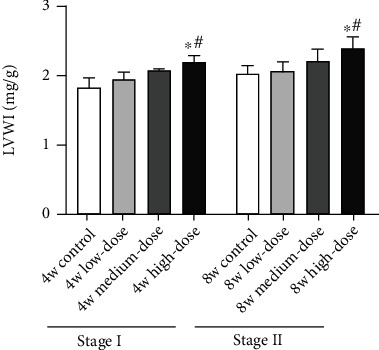
Changes in the left ventricular mass index in Stage I and Stage II rats. Notes: results are expressed as the average magnitude of each value within a group of animals ± SD (*n* = 6/group). ∗ represents *p* < 0.05 compared to the control group. # represents *p* < 0.05 compared to the low-dose group.

The LVWI of the high-dose group is significantly higher than that of the control and low-dose groups in Stage I and Stage II rats (*p* < 0.05). Compared to the control group, the medium-dose group and low-dose group exhibit an upward trend in LVWI in Stage I and Stage II rats (*p* > 0.05) (shown in Figure 1).

#### 4.2.2. Injection of MMP-9 Can Enlarge the Left Ventricle

**Figure 2:**
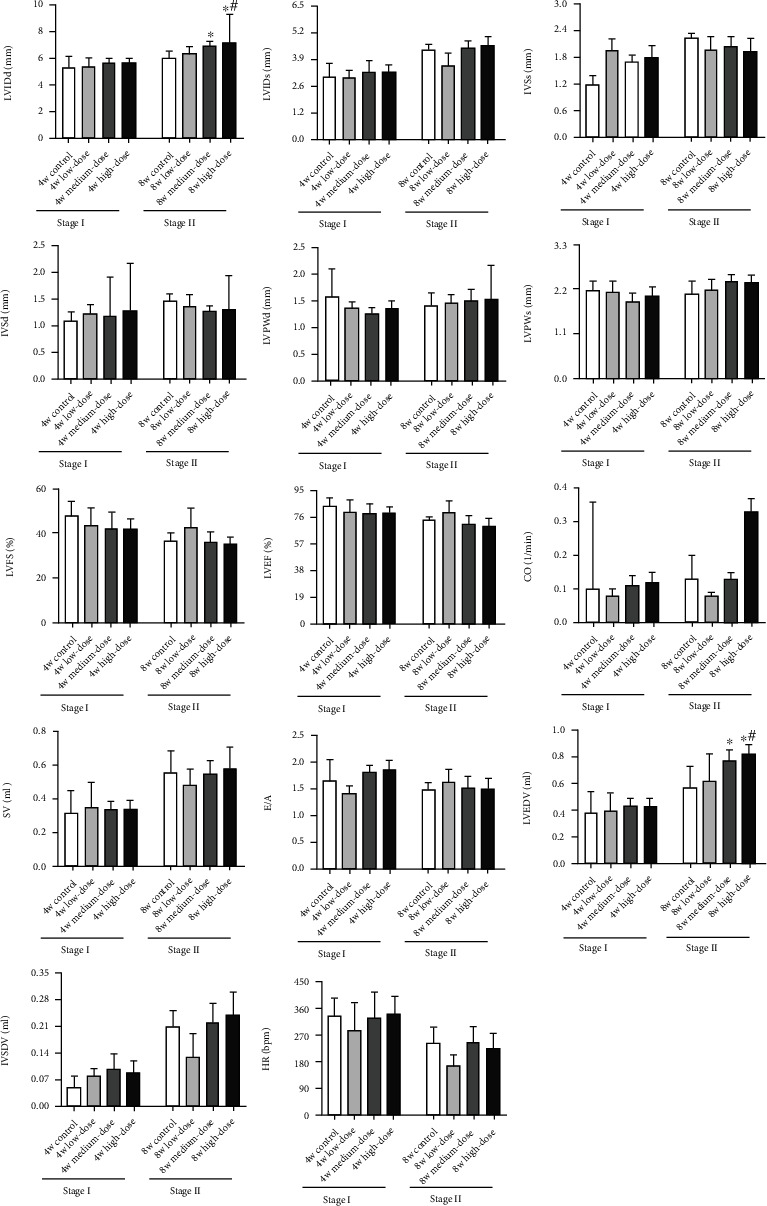
Changes in general cardiac structure and functions of Stage I and Stage II rats. Notes: LVIDd: left ventricular end-diastolic diameter; LVIDs: left ventricular end-systolic diameter; IVSd: interventricular septum diastolic diameter; IVSs: interventricular septum systole diameter; LVPWd: diastole left ventricular posterior wall diameter; LVPWs: systole left ventricular wall diameter; LVFS: left ventricular shortening fraction; LVEF: left ventricular ejection fraction; CO: cardiac output; SV stroke volume; E/A: bicuspid valve blood peak E/peak A; LVEDV: left ventricular end-diastolic volume; LVSDV: left ventricular end-systolic volume; HR: heart rate. Results are expressed as the average magnitude of each value within a group of animals ± SD (*n* = 6/group). ∗ represents *p* < 0.05 compared to the control group. # represents *p* < 0.05 compared to the low-dose group.

There are no significant differences in the cardiac ultrasonic testing indices between the various model groups in Stage I (*p* > 0.05). In Stage II, the left ventricular end-diastolic volume (LVEDV) and left ventricular end-diastolic diameter (LVIDd) of the high-dose group are far higher than those of the control and low-dose groups (*p* < 0.05). Compared to the control group, the medium-dose group exhibited significant increases in the LVEDV and LVIDd (*p* < 0.05). The posterior diastolic wall thickness (LVPWd) of the medium-dose group shows an increasing trend compared to that of the control group (*p* > 0.05). No significant differences are observed in the other indices (shown in Figure 2).

#### 4.2.3. HE Staining Shows Myocardial Damage and Structural Disorder

**Figure 3:**
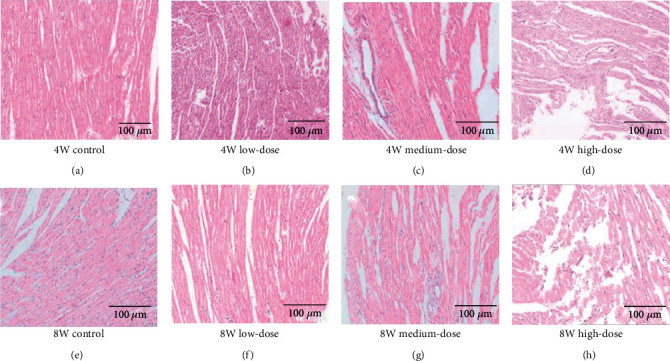
Light microscope changes in the myocardial structure of each group in Stage I and Stage II rats (HE staining, normal light microscope, ×100). In Stage I rats (a–d), compared with the control group, the medium- and high-dose groups showed pathological changes in myocardial ventricular remodeling, such as cell hypertrophy, disarrangement, cytoplasmic lysis, and necrosis. In contrast, the low-dose group had no significant change. The model observed pathological changes in ventricular remodeling in the Stage II rats (e–h) compared with the control group.

In Stage I rats, compared to the control group, the medium-dose and high-dose groups present with evident cardiomyocyte hypertrophy and disordered arrangement, accompanied by breakage of myocardial fibers, endochylema dissolution, and necrosis. There are no significant changes in the myocardial cells of the low-dose group (shown in Figures 3(a)–3(d)).

In the Stage II rats, compared to the control group, all model groups presented with breakage, damage, and myolysis of myocardial fibers, and myocardial cells had disappeared. Moreover, these lesions intensified with increasing MMP-9 levels (shown in Figures 3(e)–3(h)).

#### 4.2.4. VG Staining Shows a Reduction in Myocardial Matrix Collagen Volume Fraction (CVF)

**Figure 4:**
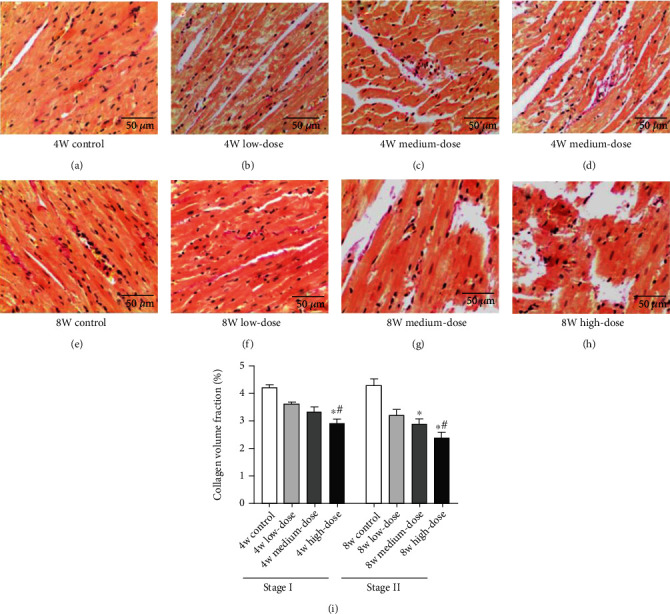
The morphology and distribution of myocardial collagen in Stage I (a–d) and Stage II (e–h) rats (VG staining, normal light microscope, ×200); (i) the CVF of the different groups in Stage I and Stage II. Notes: collagenous fibers are red, and muscle fibers are yellow. Results are expressed as the average magnitude of each value within a group of animals ± SD (*n* = 6/group). ∗ represents *p* < 0.05 compared to the control group. # represents *p* < 0.05 compared to the low-dose group.

The myocardial matrix CVF of the high-dose group decreases significantly compared to that of the control and the low-dose groups, for both Stage I and II rats (*p* < 0.05). In Stage II rats, the myocardial matrix CVF of the medium-dose group is far lower than that of the control group (*p* < 0.05). The low-dose group was not significantly different from the control group in both Stages I and II rats (*p* > 0.05) (shown in Figures 4(a)–4(i)).

#### 4.2.5. Injection of MMP-9 Can Increase Blood Pressure

**Figure 5:**
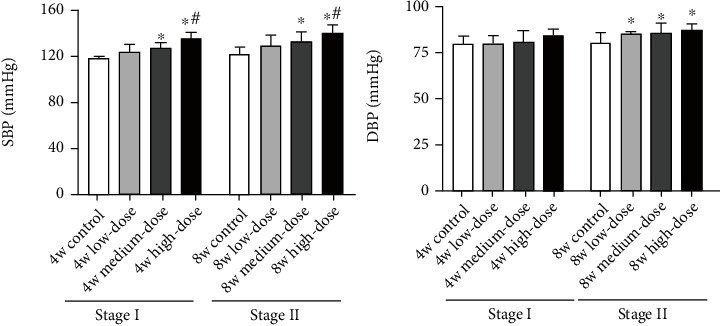
Blood pressure changes in Stage I and Stage II rats. Notes: results are expressed as the average magnitude of each value within a group of animals ± SD (*n* = 6/group). ∗ represents *p* < 0.05 compared to the control group. # represents *p* < 0.05 compared to the low-dose group.

For Stage I rats, compared with the control group, the low-dose group exhibited no significant increase in blood pressure (*p* > 0.05). In contrast, the medium- and high-dose groups show significant increases in systolic blood pressure (SBP) (*p* < 0.05). The high-dose group also has significantly higher SBP (*p* < 0.05) than the low-dose group. In the Stage II rats, the increases in SBP are similar to those in Stage I rats. SBP is significantly increased in the medium- and high-dose groups (*p* < 0.05), with a more significant increase in the high-dose group. Diastolic blood pressure (DBP) is significantly increased in all three model groups (*p* < 0.05) (shown in Figure 5).

#### 4.2.6. Injection of MMP-9 Can Increase Myocardial MMP-9, Serum MMP-9, and Myocardial MMP-9 mRNA

The high-dose group displayed significant increases in myocardial MMP-9 and serum MMP-9 compared to the control and low-dose groups, in both Stage I and II rats (*p* < 0.05). In Stage I rats, the myocardial MMP-9 mRNA of the high-dose group is significantly higher than that of the control group (*p* < 0.05) but not significantly higher than the other dose groups. There are no significant differences in myocardial MMP-9 mRNA among the groups in the Stage II rats (*p* > 0.05).

Compared to the control group, the medium-dose group shows far higher serum MMP-9 and myocardial MMP-9 mRNA in Stage I rats (*p* < 0.05). In Stage II rats, the serum MMP-9 level is further increased (*p* < 0.05), as was the myocardial MMP-9 level (*p* < 0.05), but there are no significant differences in myocardial MMP-9 mRNA among the different groups (*p* > 0.05).

**Figure 6:**
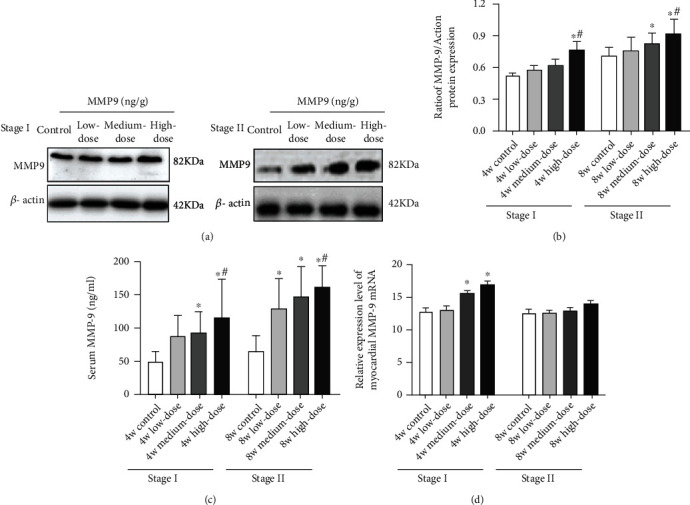
(a) Changes in the MMP-9 protein bands in myocardial tissue of the different groups. (b) Changes in MMP-9 protein levels in the myocardial tissue of each group. (c) Changes in serum MMP-9 levels in the different groups. (d) MMP-9 mRNA expression levels in the myocardial tissue of Stage I and Stage II rats. Notes: results are expressed as the average magnitude of each value within a group of animals ± SD (*n* = 6/group). ∗ represents *p* < 0.05 compared to the control group. # represents *p* < 0.05 compared to the low-dose group.

In both stages, the low-dose group presents an increasing trend in myocardial MMP-9 levels, serum MMP-9 levels, and myocardial MMP-9 mRNA compared with the control group (*p* > 0.05). The serum MMP-9 level of the low-dose group is significantly increased compared to that of the control group in Stage II rats (*p* < 0.05) (shown in Figures 6(a)–6(d)).

#### 4.2.7. Myocardial MMP-9 Levels Positively Correlated with LVWI, LVIDd, and LVEDV and Negatively Correlated with LVEF

**Figure 7:**
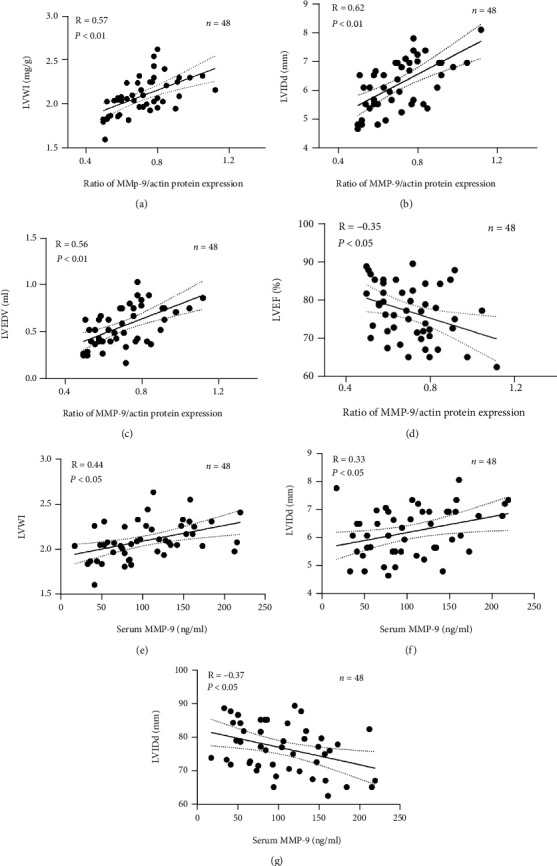
(a) Scatter plots of myocardial MMP-9 levels and LVWI. (b) Scatter plots of myocardial MMP-9 levels and LVIDd. (c) Scatter plots of myocardial MMP-9 levels and LVEDV. (d) Scatter plots of myocardial MMP-9 levels and LVEF. (e) Scatter plots of serum MMP-9 levels and LVWI. (f) Scatter plots of serum MMP-9 levels and LVIDd. (g) Scatter plots of serum MMP-9 levels and LVEF.

Correlation analysis showed that the myocardial MMP-9 level is significantly positively correlated with LVWI, LVIDd, and LVEDV, with correlation coefficients of 0.57, 0.62, and 0.56, respectively (*p* < 0.01). Myocardial MMP-9 level is negatively correlated with LVEF, with a correlation coefficient of -0.35 (*p* < 0.05) (shown in Figures 7(a)–7(d)).

#### 4.2.8. Serum MMP-9 Positively Correlated with LVWI and LVIDd and Negatively Correlated with LVEF

Correlation analysis shows that serum MMP-9 level is significantly positively correlated with LVWI and LVIDd, with correlation coefficients of 0.44 and 0.33 (*p* < 0.05), respectively. Serum MMP-9 level is negatively correlated with LVEF, with a coefficient of -0.37 (*p* < 0.05) (shown in Figures 7(e)–7(g)).

## 5. Discussion

Ventricular remodeling includes myocardial parenchymal remodeling and myocardial interstitial remodeling. Histologically, the former is characterized by cardiomyocyte hypertrophy, myocardial apoptosis, and fibroblast proliferation. The latter is characterized by extracellular matrix degradation, collagen fibrinolysis, and sedimentation. In general, it manifests as increased LVWI, myocardial hypertrophy, decreased E/A peak values of the mitral diastolic blood flow spectrum, and decreased LVEF [8]. In this study, after intraperitoneal injection of recombinant MMP-9 in healthy rats, myocardial fiber fracture, cytoplasmic lysis, and structural disorder were observed histologically. Myocardial interstitial CVF is significantly decreased. In general, increases in the ventricular mass index (LVWI), left ventricular end-diastolic volume (LVEDV), and left ventricular end-diastolic diameter (LVIDd) are observed. These findings suggest that ventricular remodeling occurred in rats after MMP-9 injection.

Further, MMP-9 and MMP-9 mRNA levels in serum and myocardial tissue are correlated with changes in the ventricular remodeling indices. The results demonstrated that the medium-dose and high-dose groups exhibited significant increases in serum MMP-9 in the fourth week. In contrast, myocardial MMP-9 is increased significantly only in the high-dose group. In the eighth week, serum MMP-9 in the medium-dose and high-dose groups is further increased. Moreover, myocardial MMP-9 in the two groups also increased significantly. These results indicate that serum MMP-9 increased earlier than myocardial MMP-9, consistent with the assertion that intraperitoneal-injected exogenous drugs are absorbed by the blood and then enter the myocardial tissue. This finding is similar to the previously reported order of elevated plasma concentrations in an in vivo model constructed by intraperitoneal injection of exogenous drugs [9, 10]. Thus, these findings indicate that the in vivo MMP-9 elevation observed here is primarily derived from exogenous sources. Secondly, in the current study, the degree of ventricular remodeling (e.g., increases in LVWI, LVEDV, LVIDd, and the degree of myocardial cell destruction and decreases in myocardial interstitial CVF) increased in a dose- and time-dependent manner with MMP-9 injections. Regarding the correlations between the ventricular remodeling indices and the myocardial tissue and serum MMP-9 levels, it is noted that myocardial MMP-9 was significantly higher in the high-dose group in the fourth week. However, the changes to the ventricular remodeling indices, such as LVWI, LVEDV, LVIDd, myocardial cells, and interstitium, were not sufficiently noticeable at this time. The relevant indicators of ventricular remodeling did not exhibit statistically significant changes until the eighth week. The lag in myocardial remodeling behind the elevation in MMP-9 is consistent with the temporal characteristics of MMP-9 as the cause of ventricular remodeling.

Moreover, myocardial and serum MMP-9 levels correlate significantly and positively with LVWI, LVIDd, and LVEDV. LVEF is significantly negatively correlated with myocardial and serum MMP-9. The myocardial MMP-9 level correlates better with the ventricular remodeling parameters than the serum MMP-9 levels. This further supports the assertion that myocardial remodeling is related to the local myocardial MMP-9 elevation. However, the correlation between LVEF and MMP-9 is lower than the correlations for the above cardiac structural indicators, an index considered more closely related to structural changes in early ventricular remodeling than functional decline. In addition, the results of this study indicate that MMP-9 injections resulted in MMP-9 mRNA elevation, suggesting the induction of endogenous MMP-9 expression. However, this phenomenon was only observed in the first-stage group and not in the second-stage group, suggesting that the induction of endogenous MMP-9 expression is transient and does not last long. This outcome also suggests that the further increase in myocardial MMP-9 in the second-stage group was mainly due to continuous exogenous input. Finally, this study's finding of ventricular remodeling after MMP-9 injection is consistent with previously reported ventricular remodeling with elevated MMP-9 characteristics and the action of MMP-9 itself [4].

As a proteolytic enzyme, MMP-9 can act on several substrates in the extracellular matrix, including cleaved collagen, thereby decreasing collagen content [4]. MMP-9 can also induce cardiomyocyte hypertrophy, apoptosis, death, and myocardial myosin heavy chain rupture, damaging the myocardial structure and contractile ability [11–13]. After injection of MMP-9, the high MMP-9 ventricular remodeling (LVR-hMMP-9) model rats exhibited myocardial cell damage, reductions in CVF, disordered arrangement, and expansion of cardiac chambers, consistent with the characteristics of MMP-9 ventricular remodeling. There was also a significant increase in blood pressure after MMP-9 injection, and hypertension can also lead to ventricular remodeling.

The main manifestations of hypertension-induced ventricular remodeling are increased collagen fiber content in the interstitium, ventricular wall hypertrophy, and reduced left ventricular diameter [14]. However, in the current model, CVF decreased and left ventricular diameter increased. Therefore, although blood pressure is significantly increased in the model group, elevated blood pressure does not seem to be the primary cause of ventricular remodeling. The correlations between the myocardial MMP-9 level and the ventricular remodeling indices also further support the assertion that ventricular remodeling in this model was mainly caused by MMP-9. However, the mechanisms underlying the MMP-9-induced increase in blood pressure and the resulting effect on ventricular remodeling deserve further study.

The above characteristics of ventricular remodeling after MMP-9 infusion are consistent with previously reported characteristics of ventricular remodeling with increased MMP-9 [8, 13]. In the current study, the increases in ventricular remodeling indicators were not only positively correlated with increases in myocardial and serum MMP-9 levels, but these effects were also time- and dose-dependent. Such characteristics further support the assertion that MMP-9 is a causal factor in ventricular remodeling.

The current study examined an experimental ventricular remodeling model that resulted from infusion of exogenous MMP-9 rather than examining MMP-9 ventricular remodeling in a disease state (for example, experimental myocardial infarction [15–17] and tachycardia cardiomyopathy caused by a cardiac pacemaker [18, 19]) or caused by the injection of other drugs [20–22] (such as adriamycin [23, 24]). Therefore, the results are not affected by a primary disease or the confounding effects of other drugs. Such independence provides further confidence that the ventricular remodeling observed in this study was directly caused by elevated MMP-9.

The findings of this study not only provide direct and unmistakable evidence that MMP-9 leads to ventricular remodeling but also validate a new and straightforward method to construct a ventricular remodeling model for future studies. The ventricular remodeling performance of the model developed in this study met the previously reported success criteria for ventricular remodeling models [8]. Thus, this model can serve as a new model of LVR-hMMP-9. The increased MMP-9 and/or MMP-9 mRNA expression and histopathological remodeling changes observed with the current model are consistent with the characteristics of MMP-9 tissues [13].

From the perspective of model construction, the described modeling method also has the following advantages. First, the model rats had a high survival rate, as the survival rate of the model rats was 100%, and the survival period was prolonged. Body weight did not fluctuate significantly. The mortality rates of previous models based on coronary occlusion range from 7 to 25%, and the mortality during the perioperative period is relatively high due to the influence of coronary ligation techniques, anesthetics, and ventilator intubation for small animals [25, 26]. The mortality of modeling based on isoproterenol injection is reported to reach 16-50% [20, 27, 28]^,^ and the survival period of the animals is short, 12.5 days on average [28]. The mortality of modeling based on adriamycin is reported to reach 30%, accompanied by a reduction in body weight [24]. Animal models are powerful tools to evaluate the pathological mechanisms of diseases and the therapeutic effects of drugs. However, short survival periods, high mortality, and significant fluctuations in the body weight of model animals increase the uncertainty of the research outcomes, thus influencing the reliability of the final research conclusions.

Secondly, the current model is easy to generate. Modeling based on rapid cardiac pacing requires cardiac pacing equipment and the catheter technique [18, 19]. While the catheter technique generally applies to large animals, it is more difficult to implant cardiac pacing wires into mice or rats. The proposed model induces ventricular remodeling to different degrees, by controlling the drug dosage and enabling good modeling control. Such control differs from modeling based on coronary occlusion or ligation, which requires accurate anatomical positioning. In particular, the structure of the rat coronary artery cannot be easily distinguished with the naked eye, resulting in difficulty determining the branches of the coronary artery and the ideal ligation position [15–17]. In previous studies, the infarct size in mice fluctuates between 8 and 65% due to different ligation positions and degrees. Moreover, infarct size is the main decisive factor in left ventricular remodeling and mortality [29, 30]. Therefore, difficulty with accurately controlling the infarct size might influence the model consistency. The directional gene high expression method can also be used to construct a high MMP ventricular remodeling model and has good etiological directivity. However, it requires complex genetic engineering techniques for induction. In addition, there may be extensive differences between individuals in the MMP protein expression levels, which may be slightly less optimal for studying the relationship between the amount of MMP-9 and the degree of ventricular remodeling, as compared to our current model. The proposed LVR-hMMP-9 model involves intraperitoneal injection and does not require advanced instruments or high-precision equipment. Researchers only need to master the simple intraperitoneal injection method, making this model easy to establish.

## 6. Conclusions and Prospects

The current study demonstrates that injection of MMP-9 can lead to ventricular remodeling. This study also describes a relatively simple method to establish a high-MMP-9 ventricular remodeling model, which can serve as a good research tool for studying the mechanism of ventricular remodeling, developing therapeutic targets, and evaluating the therapeutic effects of new drugs. However, the model did not produce significant anomalies in some cardiac functions, including LVEF and E/A, potentially due to the short experimental period of this study. As a model construction method, it is still necessary to further explore the appropriate dose and time of MMP-9 injection for an optimum model, as well as the function of the right ventricle, left atrium, and essential organs such as the liver, kidneys, and lungs. Finally, the mechanism underlying the MMP-9-induced increase in blood pressure and its influence on cardiovascular remodeling must be further investigated and understood.

## Figures and Tables

**Table 1:** Body weights and blood pressure measurements.

	Stage I (*n* = 24)			Stage II (*n* = 24)		
	Initial weight (g)	Initial SBP (mmHg)	Initial DBP (mmHg)	Initial weight (g)	Initial SBP (mmHg)	Initial DBP (mmHg)
Control	207 ± 16.7	121 ± 7.8	81 ± 4.6	205 ± 19.9	124 ± 9.4	80 ± 6.0
Low dose	206 ± 16.8	123 ± 5.8	81 ± 5.0	207 ± 14.5	122 ± 8.4	81 ± 4.5
Medium dose	210 ± 14.8	120 ± 6.4	79 ± 6.5	205 ± 18.4	121 ± 7.3	79 ± 5.2
High dose	209 ± 14.3	118 ± 8.4	78 ± 6.2	209 ± 17.3	119 ± 9.7	80 ± 4.9

Notes: data are presented as the mean ± SD. SBP: systolic pressure; DBP: diastolic pressure.

## Data Availability

All data generated or analyzed during this study are included in this article. Further enquiries can be directed to the corresponding author.
